# Preoperative prognostic model combining tumor burden score and tumor markers to predict long-term outcomes following hepatectomy for intrahepatic cholangiocarcinoma: a multi-institutional analysis

**DOI:** 10.3389/fonc.2026.1720482

**Published:** 2026-02-11

**Authors:** Jun Fu, Tingfeng Huang, Qizhu Lin, Jingdong Li, Xinyu Bi, Jianming Wang, Fuyu Li, Jian Wang, Kui Wang, Jianying Lou, Shi Cheng, Yongyi Zeng

**Affiliations:** 1 Department of Hepatopancreatobiliary Surgery, First Affiliated Hospital of Fujian Medical University, Fuzhou, Fujian, China; 2 Department of Hepatobiliary Surgery, Mengchao Hepatobiliary Hospital of Fujian Medical University, Fuzhou, Fujian, China; 3 Department of Hepatobiliary Surgery, The Affiliated Hospital of Chuanbei Medical University, Nanchong, Sichuan, China; 4 Department of Hepatobiliary Surgery, Cancer Hospital, Chinese Academy of Medical Sciences, Beijing, China; 5 Department of Hepatobiliary Surgery, Tongji Hospital Affiliated to Tongji Medical College, Huazhong University of Science Technology, Wuhan, Hubei, China; 6 Department of Hepatobiliary Surgery, The West China Hospital of Sichuan University, Chengdu, Sichuan, China; 7 Department of Hepatobiliary Surgery, Renji Hospital Affiliated to Shanghai Jiaotong University, Shanghai, China; 8 Department of Hepatic Surgery (II), Eastern Hepatobiliary Surgery Hospital, Navy Medical University, Shanghai, China; 9 Department of Hepatobiliary Surgery, The Second Hospital Affiliated to Zhejiang University, Hangzhou, Zhejiang, China; 10 Department of Hepatobiliary Surgery, Tiantan Hospital Affiliated to Capital Medical University, Beijing, China

**Keywords:** adjuvant chemotherapy, carbohydrate antigen 19-9 (CA 19-9), carcinoembryonic antigen (CEA), preoperative risk model, tumor burden score (TBS)

## Abstract

**Background and aim:**

Intrahepatic cholangiocarcinoma (ICC) is an aggressive liver malignancy with limited prognostic tools to guide treatment strategies. This study aimed to develop and validate a preoperative prognostic model combining tumor burden score (TBS), carcinoembryonic antigen (CEA), and carbohydrate antigen 19-9 (CA19-9), termed the TCCA model, to predict outcomes in patients with ICC undergoing hepatectomy.

**Methods:**

Patients who underwent curative resection for ICC between 2014 and 2020 were retrospectively identified from a multi-institutional database. The impact of the TCCA model on overall survival (OS) and recurrence-free survival (RFS) was evaluated in training and validation cohorts. Predictive performance was evaluated using the area under the Receiver Operating Characteristic curve (AUC), the Akaike Information Criterion (AIC), and the C-index.

**Results:**

A total of 849 patients were included. Lower TCCA scores were associated with better median OS (score 0: 59.7 months; score 1: 31.3 months; score 2: 19.4 months; score 3: 11.5 months, respectively) and median RFS (28.8; 15.4; 9.7; 8.1 months, respectively). The TCCA model performed well in both the training cohort (AUC: 0.697 for OS and 0.649 for RFS) and the validation cohort (AUC: 0.672 for OS and 0.632 for RFS), outperforming the 8th edition TNM system and other models, with the highest C-index (0.734) and lowest AIC (3840). Subgroup analyses demonstrated that the TCCA model maintained good discriminative ability among patients with negative CEA or CA19–9 levels.

**Conclusion:**

The TCCA model accurately stratifies ICC patients for OS and RFS after resection. It provides a simple and practical tool for preoperative risk assessment, supporting individualized surgical decision-making and individualized patient counseling.

## Introduction

Intrahepatic cholangiocarcinoma (ICC), the second most common primary liver cancer, arises from the epithelium lining the peribiliary glands of secondary or higher-order bile ducts (1). Despite an increase in incidence over recent decades, ICC remains a rare malignancy, with most institutions performing few resections annually (2). Although radical resection is the most effective treatment for resectable ICC, the disease has a high propensity for recurrence and metastasis rate, leading to poor long-term outcomes, with reported 5-year overall survival (OS) rates ranging from 20% to 40% (3).

Even when standard criteria for resectable ICC are applied, it remains unclear which subgroup of patients truly benefits from surgical resection in terms of long-term survival (4). To better stratify patients undergoing resection, the American Joint Committee on Cancer (AJCC) regularly updates the Tumor−Node−Metastasis (TNM) classification system (5). However, the TNM classification system relies on postoperative pathological data, limiting its utility for preoperative decision-making (6). Several postoperative prognostic models have also been proposed, but they share similar limitations (7, 8). Given the aggressive nature of ICC, accurate preoperative prognostic evaluation is crucial for optimizing treatment strategies.

Various preoperative prognostic models have been introduced to improve treatment decision-making for ICC, incorporating clinical parameters such as radiologic features and inflammatory markers (9–12). While these models show promise, many lack sufficient accuracy or are too complex for routine clinical practice. For instance, radiomics-based models, although effective, require advanced imaging analysis tools that may not be readily available in routine clinical practice (13). Additionally, models based on inflammatory markers, such as Neutrophil−to−Lymphocyte Ratio (NLR), Prognostic Nutritional Index (PNI), however, may fail to capture the full oncologic complexity of ICC, underscoring the need for a more balanced yet comprehensive scoring system that is both practical and accurate in predicting patient outcomes (14, 15).

The tumor burden score (TBS), which combines tumor size and number, is a well-established prognostic factor for ICC and is now part of the AJCC-T classification. Moreover, elevated serum levels of carcinoembryonic antigen (CEA) or Carbohydrate antigen 19-9 (CA19-9) are associated with adverse outcomes and reflect underlying oncological behavior (16). However, previous studies have generally combined TBS with a single biomarker such as CA19–9 or CEA (17, 18). Relying on one serum marker may underestimate the biological heterogeneity of ICC, particularly in patients who are CA19-9- or CEA-negative.

To the best of our knowledge, this study is the first to integrate TBS, CEA, and CA19–9 into a single preoperative model—termed the TCCA model—to provide a more comprehensive and balanced assessment of both tumor burden and tumor biology, thereby improving individualized risk stratification and treatment planning for patients with ICC undergoing liver resection.

## Methods

### Patients and selection criteria

The retrospective study collected data from nine large tertiary medical institutions across China between 2014 and 2020. The training cohort data were obtained from Mengchao Hepatobiliary Hospital of Fujian Medical University, Eastern Hepatobiliary Surgery Hospital of Naval Medical University, and The Affiliated Hospital of Chuanbei Medical University. The validation cohort data were gathered from Cancer Hospital of the Chinese Academy of Medical Sciences, Tongji Hospital Affiliated to Tongji Medical College, Renji Hospital Affiliated to Shanghai Jiaotong University, The Second Hospital Affiliated to Zhejiang University, Tiantan Hospital Affiliated to Capital Medical University, and West China Hospital of Sichuan University. Informed consent was obtained from all patients before surgery, adhering strictly to the guidelines of the Declaration of Helsinki. Ethical approval was obtained from the Institutional Review Boards of all participating institutions (approval number 2023_017_01).

The inclusion criteria were as follows: (1) ECOG score of 0−2, (2) Child-Pugh score between A5 to B7, (3) Dynamic contrast-enhanced computed tomography (CT) or Multi-phase magnetic resonance imaging (MRI) assessment of tumor size and number within one month before surgery, (4) serum levels of CEA and CA19–9 were measured within one week prior to surgery, (5) R0 resection with postoperative pathological confirmation of ICC. The exclusion criteria were as follows: (1) receipt of preoperative therapies such as radiofrequency ablation, local interventional procedures, or chemotherapy, (2) recurrent ICC or other simultaneously malignancies, (3) died within 30 days or were lost to follow−up within 3 months postoperatively.

### Variables of interested

Demographic and preoperative laboratory examination data were collected, including age, gender, Hepatitis B surface antigen (HBsAg), presence of cirrhosis, Child-Pugh grade, as well as CA19–9 and CEA levels. Inflammatory and nutritional markers such as NLR, platelet-to-lymphocyte ratio (PLR), PNI, andγ-glutamyl transferase to alanine aminotransferase ratio (GAR) were also calculated, and determine the optimal cutoff values based on ROC analysis. Number of tumors and the size of the largest tumor were assessed via CT or MRI. Additional surgical and pathological data included details on the anatomic resection, margin width, tumor differentiation, satellite nodular, macrovascular and microvascular invasion, as well as information on postoperative adjuvant chemotherapy. TNM staging followed the 8^th^ edition of the AJCC staging manual.

### Definition of TBS and TCCA model

TBS is determined as the Euclidean distance on a Cartesian plane, based on two factors: the largest tumor size (x-axis) and the total number of tumors (y-axis). In cases with multiple lesions, the tumor size refers to the largest nodule. The formula for calculating TBS is TBS² = (tumor size)² + (number of tumors)² (19). For example, if the largest tumor measures 3 cm and there are four tumors in total, the TBS can be calculated as TBS = √(3² + 4²) = 5, resulting in a TBS value of 5 units.

The TCCA model is composed of three preoperative variables derived from multivariate analysis: TBS, CEA, and CA19-9. Each continuous variable is dichotomized into low and high groups. The optimal critical value of TBS was determined to be 6.0 units through ROC analysis. The cutoff values for CEA (5.0 ng/mL) and CA19-9 (37.0 U/mL) are based on the normal upper limit for laboratory examination. Patients are assigned a score of 1 for each high group and 0 for each low group, resulting in a total TCCA score ranging from 0 to 3 points.

### Definition of other important clinical and follow−up related variables

Microvascular invasion (MVI) was defined as intraparenchymal vascular involvement, while macrovascular invasion involved major branches of the portal vein, hepatic artery, or hepatic veins, all based on histological examination. Adjuvant chemotherapy includes the administration of gemcitabine-based chemotherapy regimens or capecitabine-based monotherapy within 1 to 2 months postoperative, with or without TACE. The primary outcome was OS, defined as the interval from ICC resection to death or last follow-up, while the secondary outcome was recurrence-free survival (RFS), defined as the time from resection to recurrence or last follow-up. Recurrence was confirmed by tumor biopsy or follow-up imaging identifying suspicious lesions.

### Statistical analysis

Continuous variables were summarized as mean ± standard deviation (SD) or median with interquartile range (IQR). Group differences were evaluated using one-way ANOVA or the Kruskal-Wallis tests. Categorical variables were reported as frequencies and percentages, with group comparisons conducted using the Chi-square test. The ROC curve, Harrell c-index, and Akaike information criterion (AIC) were used to assess the discriminative ability and predictive accuracy of the model, while Kaplan-Meier survival analysis was conducted to evaluate long-term outcomes. Model calibration was assessed using calibration plots and the Hosmer-Lemeshow test. Statistical analysis was executed using SPSS^®^ version 25.0 (IBM, Armonk, New York, USA) and R program version 3.2.0 (http://www.r-project.org/). A p-value of less than 0.05 was deemed statistically significant.

## Results

### Baseline characteristics of training and validation cohorts

A total of 849 ICC patients were retrospectively reviewed, including 635 in the training cohort and 214 in the validation cohort. As summarized in **Table 1**, the mean age of the entire cohort was 54.4 years, with the majority being male (n =576, 67.8%). Approximately half of the patients were HBsAg-positive (n =411, 48.4%). The mean maximum tumor size was 6.25 cm, and 16.6% of patients had multiple tumors. Compared to the validation cohort, the training cohort had higher proportions of liver cirrhosis (31.0% *vs.* 23.4%), elevated CEA levels (28.3% *vs.* 20.0%), MVI (14.2% *vs.* 8.4%), and poorer tumor differentiation (93.7% *vs.* 88.3%), while fewer patients received postoperative adjuvant chemotherapy (12.8% *vs.* 20.6%). There was no significant difference in CA19–9 levels between the two cohorts.

**Table 1:** Baseline clinicopathological characteristics of the training and validation cohort.

Variables (%)	level	Overall	Training group	Validation group	*P* value
		849(100%)	635(74.8%)	214(25.2%)	
Age, year*		54.5 ± 10.8	54.4 ± 10.9	55.0 ± 10.6	0.584
Sex (%)	female	273 (32.2)	195 (30.7)	78 (36.5)	0.142
	male	576 (67.8)	440 (69.3)	136 (63.5)	
HBsAg (+) (%)	no	438 (51.6)	328 (51.7)	110 (51.4)	1
	yes	411 (48.4)	307 (48.3)	104 (48.6)	
Cirrhosis (%)	no	602 (70.9)	438 (69.0)	164 (76.6)	**0.041**
	yes	247 (29.1)	197 (31.0)	50 (23.4)	
Child-Pugh grade (%)	A	803 (94.6)	606 (95.4)	197 (92.1)	0.087
	B	46 (5.4)	29 (4.6)	17 (7.9)	
NLR*		3.23 ± 3.40	3.28 ± 3.80	3.07 ± 1.75	0.421
PLR*		139.9 ± 171.3	143.0 ± 193.7	130.813 ± 70.5	0.368
PNI*		50.4 ± 6.1	50.5 ± 6.3	49.9 ± 5.4	0.226
GAR*		4.22 ± 5.66	4.37 ± 6.17	3.78 ± 3.74	0.186
CEA, ng/mL (%)	≤5.0	626 (73.7)	455 (71.7)	171 (80.0)	**0.022**
	>5.0	223 (26.3)	180 (28.3)	43 (20.0)	
CA19-9, U/mL (%)	≤37.0	405 (47.7)	306 (48.2)	99 (46.3)	0.683
	>37.0	444 (52.3)	329 (51.8)	115 (53.7)	
Maximum tumor size, cm*		6.25 ± 3.0	6.21 ± 3.0	6.34 ± 3.0	0.589
Tumor number (%)	single	708 (83.4)	533 (84.0)	175 (81.8)	0.530
	multiple	141 (16.6)	102 (16.0)	39 (18.2)	
Anatomic resection (%)	no	203 (23.9)	160 (25.2)	43 (20.1)	0.155
	yes	646 (76.1)	475 (74.8)	171 (79.9)	
Margin width, cm (%)	<1	527 (62.1)	386 (60.8)	141 (65.9)	0.212
	≥1	322 (37.9)	249 (39.2)	73 (34.1)	
Macrovascular invasion (%)	No	733 (86.3)	547 (86.1)	186 (86.9)	0.865
	yes	116 (13.7)	88 (13.9)	28 (13.1)	
Microvascular invasion (%)	no	741 (87.3)	545 (85.8)	196 (91.6)	**0.039**
	yes	108 (12.7)	90 (14.2)	18 (8.4)	
Satellite nodules (%)	no	634 (74.7)	482 (75.9)	152 (71.0)	0.184
	yes	215 (25.3)	153 (24.1)	62 (29.0)	
Tumor differentiation (%)	well	65 (7.7)	40 (6.3)	25 (11.7)	**0.016**
	poor to moderate	784 (92.3)	595 (93.7)	189 (88.3)	
Lymph node status (%)	N0 + Nx	667 (78.6)	493 (77.6)	174 (81.3)	0.301
	N1	182 (21.4)	142 (22.4)	40 (18.7)	
AJCC 8^th^ edition staging system (%)	I	416 (49.00)	307 (48.35)	109 (50.93)	0.295
	II	221 (26.03)	161 (25.35)	60 (28.04)	
	III	212 (24.97)	167 (26.30)	45 (21.03)	
Adjuvant chemotherapy (%)	no	724 (85.3)	554 (87.2)	170 (79.4)	**0.008**
	yes	125 (14.7)	81 (12.8)	44 (20.6)	

HBsAg, Hepatitis B surface antigen; NLR, neutrophil-to-lymphocyte ratio; PLR, platelet-to-lymphocyte ratio; PNI, prognostic nutritional index; GAR, γ−glutamyl transferase to alanine aminotransferase ratio; CA 19-9, carbohydrate antigen 19-9; CEA, carcinoembryonic antigen; AJCC, American Joint Committee on Cancer.

*Values are mean ± standard deviation or median (interquartile range) unless otherwise indicated.

Bold values indicate p < 0.05.

A total of 635 patients were classified into four TCCA score groups (0 to 3) in the training cohort as summarized in **Table 2**. The mean age increased from 53.0 to 56.1 years as rising TCCA score, though the differences were not statistically significant (*p* = 0.068). There was also a non-significant trend towards more female patients in the higher TCCA groups (*p* = 0.101). Higher TCCA scores correlated with a decreased prevalence of HBsAg-positive status, elevated NLR, GAR, CEA, CA19-9, TBS, and a higher prevalence of macrovascular invasion, satellite nodules, lymph node metastasis, and advanced TNM stage (*p* < 0.05 for all). Additionally, patients with higher TCCA scores had larger tumors, underwent more anatomical resections, but had narrower resection margins and received more adjuvant chemotherapy (*p* < 0.05 for all).

**Table 2:** Demographic and clinical characteristics by TCCA score (0 to 3) in the training cohort.

Variables (%)	level	0	1	2	3	*P* value
		153(24.1%)	231(36.4%)	166(26.1%)	85(13.4%)	
Age, year*		53.0 ± 10.7	53.8 ± 10.3	55.5 ± 11.2	56.1 ± 11.6	0.068
Sex (%)	female	39 (25.5)	63 (27.3)	55 (33.1)	33 (38.8)	0.101
	male	114 (74.5)	168 (72.7)	111 (66.9)	52 (61.2)	
HBsAg (+) (%)	no	57 (37.3)	124 (53.7)	92 (55.4)	55 (64.7)	**<0.001**
	yes	96 (62.7)	107 (46.3)	74 (44.6)	30 (35.3)	
Cirrhosis (%)	no	94 (61.4)	162 (70.1)	116 (69.9)	66 (77.6)	0.064
	yes	59 (38.6)	69 (29.9)	50 (30.1)	19 (22.4)	
Child-Pugh grade (%)	A	147 (96.1)	216 (93.5)	163 (98.2)	80 (94.1)	0.148
	B	6 (3.9)	15 (6.5)	3 (1.8)	5 (5.9)	
NLR*		2.60 ± 2.03	3.14 ± 2.21	3.89 ± 6.26	3.717 ± 3.04	**0.014**
PLR*		111.6 ± 47.0	154.9 ± 233.5	156.2 ± 249.3	141.3 ± 73.5	0.128
PNI*		51.6 ± 7.7	50.5 ± 5.5	50.2 ± 5.9	49.2 ± 6.1	**0.040**
GAR*		2.42 ± 2.47	3.96 ± 3.84	5.73 ± 8.70	6.33 ± 8.50	**<0.001**
CEA, ng/mL (%)	≤5.0	153 (100.0)	210 (90.9)	92 (55.4)	0 (0.0)	**<0.001**
	>5.0	0 (0.0)	21 (9.1)	74 (44.6)	85 (100.0)	
CA19-9, U/mL (%)	≤37.0	153 (100.0)	127 (55.0)	26 (15.7)	0 (0.0)	**<0.001**
	>37.0	0 (0.0)	104 (45.0)	140 (84.3)	85 (100.0)	
Maximum tumor size, cm*		3.84 ± 1.2	6.00 ± 3.0	7.36 ± 2.9	8.84 ± 2.3	**<0.001**
Tumor number (%)	single	134 (87.6)	191 (82.7)	138 (83.1)	70 (82.4)	0.569
	multiple	19 (12.4)	40 (17.3)	28 (16.8)	15 (17.6)	
TBS, units (%)	≤6.0	153 (100.0)	125 (54.1)	48 (28.9)	0 (0.0)	**<0.001**
	>6.0	0 (0.0)	106 (45.9)	118 (71.1)	85 (100.0)	
Anatomic resection (%)	no	53 (34.6)	64 (27.7)	33 (19.9)	10 (11.8)	**<0.001**
	yes	100 (65.4)	167 (72.3)	133 (80.1)	75 (88.2)	
Margin width, cm (%)	<1.0	71 (46.4)	143 (61.9)	104 (62.7)	68 (80.0)	**<0.001**
	≥1.0	82 (53.6)	88 (38.1)	62 (37.3)	17 (20.0)	
Macrovascular invasion (%)	no	142 (92.8)	201 (87.0)	136 (81.9)	68 (80.0)	**0.012**
	yes	11 (7.2)	30 (13.0)	30 (18.1)	17 (20.0)	
Microvascular invasion (%)	no	136 (88.9)	204 (88.3)	138 (83.1)	67 (78.8)	0.080
	yes	17 (11.1)	27 (11.7)	28 (16.9)	18 (21.2)	
Satellite nodules (%)	no	129 (84.3)	180 (77.9)	114 (68.7)	59 (69.4)	**0.004**
	yes	24 (15.7)	51 (22.1)	52 (31.3)	26 (30.6)	
Tumor differentiation (%)	well	15 (9.8)	12 (5.2)	10 (6.0)	3 (3.5)	0.188
	poor to moderate	138 (90.2)	219 (94.8)	156 (94.0)	82 (96.5)	
Lymph node status (%)	N0 + Nx	137 (89.5)	182 (78.8)	113 (68.1)	61 (71.8)	**<0.001**
	N1	16 (10.6)	49 (21.2)	53 (31.9)	24 (28.2)	
AJCC 8^th^ edition staging system (%)	I	100 (65.3)	111 (48.1)	64 (38.6)	32 (37.6)	**<0.001**
	II	35 (22.9)	62 (26.8)	45 (27.1)	19 (22.4)	
	III	18 (11.8)	58 (25.1)	57 (34.3)	34 (40.00)	
Adjuvant chemotherapy (%)	no	142 (92.8)	203 (87.9)	142 (85.5)	67 (78.8)	**0.017**
	yes	11 (7.2)	28 (12.1)	24 (14.5)	18 (21.2)	

HBsAg, Hepatitis B surface antigen; NLR, neutrophil-to-lymphocyte ratio; PLR, platelet-to-lymphocyte ratio; PNI, prognostic nutritional index; GAR, γ−glutamyl transferase to alanine aminotransferase ratio; CA 19-9, carbohydrate antigen 19-9; CEA, carcinoembryonic antigen; AJCC, American Joint Committee on Cancer. TBS, Tumor Burden Score; TCCA, TBS, CEA, and CA19–9 combined score.

*Values are mean ± standard deviation or median (interquartile range) unless otherwise indicated.

Bold values indicate p < 0.05.

### Independent prognostic factors for OS and RFS in the training cohorts

In the multivariate Cox model for OS in the training cohort, significant independent predictors included GAR >6.46 (HR: 1.36, 95% CI: 1.05−1.78, *p* = 0.022), CEA >5.0 ng/mL (HR: 1.42, 95% CI: 1.13−1.78, *p* = 0.003), CA19-9 >37 U/mL (HR: 1.46, 95% CI: 1.16−1.83, *p* = 0.001), TBS >6.0 units (HR: 1.68, 95% CI: 1.35−2.11, *p* < 0.001), macrovascular invasion (HR: 1.54, 95% CI: 1.16−2.04, *p* = 0.003), satellite nodules (HR: 1.36, 95% CI: 1.08−1.73, *p* = 0.010), and lymph node status (N1 *vs.* N0/Nx: HR: 1.73, 95% CI: 1.34−2.23, *p* < 0.001) (**Table 3**). For RFS, the independent predictors were GAR >6.46 (HR: 1.30, 95% CI: 1.03−1.70, *p* = 0.028), CEA >5.0 ng/mL (HR: 1.25, 95% CI: 1.01−1.56, *p* = 0.042), CA19-9 >37 U/mL (HR: 1.47, 95% CI: 1.19−1.81, *p* < 0.001), TBS >6.0 units (HR: 1.55, 95% CI: 1.26−1.91, *p* < 0.001), and lymph node status (N1 *vs.* N0/Nx: HR: 1.63, 95% CI: 1.29−2.06, *p* < 0.001) (**Table 4**).

**Table 3:** Univariable and multivariable analyses for OS in the training cohort.

Variables	HR comparison	UV HR (95% CI)	UV *p* value	MV HR (95% CI)	MV *P* value
Age, year	≥60 *vs.* < 60	1.20 (0.97−1.49)	0.006	1.19(0.94−1.50)	0.138
Sex	Male *vs.* Female	0.80 (0.64−0.99)	0.048	0.82(0.65−1.02)	0.080
HBsAg	Positive *vs.* Negative	0.75 (0.60−0.92)	0.006	1.02(0.81−1.30)	0.845
Cirrhosis	Yes *vs.* No	0.82 (0.65−1.04)	0.097		
Child-Pugh grade	B *vs.* A	1.22 (0.73−2.05)	0.447		
NLR	>2.15 *vs.* ≤2.15	1.70 (1.35−2.14)	<0.001	1.21(0.94−1.57)	0.145
PLR	>115.1 *vs.* ≤115.1	1.48 (1.20−1.83)	<0.001	1.01(0.79−1.29)	0.948
PNI	>53.15 *vs.* ≤53.15	0.97 (0.95−0.99)	0.002	0.99(0.97−1.01)	0.381
GAR	>6.46 *vs.* ≤6.46	2.04 (1.59−2.60)	<0.001	1.36(1.05−1.78)	**0.022**
CEA, ng/mL	>5.0 *vs.* ≤5.0	1.87 (1.50−2.33)	<0.001	1.42(1.13−1.78)	**0.003**
CA19-9, U/mL	>37.0 *vs.* ≤37.0	1.92 (1.55−2.37)	<0.001	1.46(1.16−1.83)	**0.001**
TBS, units	>6.0 *vs.* ≤6.0	2.13 (1.72−2.63)	<0.001	1.68(1.35−2.11)	**<0.001**
Anatomic resection	Yes *vs.* No	1.23 (0.97−1.58)	0.093		
Margin width, cm	≥1.0 *vs.* ≤1.0	0.73 (0.59−0.91)	0.004	0.89(0.71−1.12)	0.307
Macrovascular invasion	Yes *vs.* No	1.78 (1.35−2.34)	<0.001	1.54(1.16−2.04)	**0.003**
Microvascular invasion	Yes *vs.* No	1.50 (1.14−1.97)	0.004	1.21(0.91−1.61)	0.186
Satellite nodules	Yes *vs.* No	1.77 (1.41−2.22)	<0.001	1.36(1.08−1.73)	**0.010**
Tumor differentiation	Poor to moderate *vs.* Well	1.04 (0.68−1.60)	0.854		
Lymph node status	N1 *vs.* N0 + Nx	2.21 (1.75−2.8)	<0.001	1.73(1.34−2.23)	**<0.001**
Adjuvant chemotherapy	Yes *vs.* No	0.87 (0.64−1.19)	0.377		

HBsAg, Hepatitis B surface antigen; NLR, neutrophil-to-lymphocyte ratio; PLR, platelet-to-lymphocyte ratio; PNI, prognostic nutritional index; GAR, γ−glutamyl transferase to alanine aminotransferase ratio; CA19-9, carbohydrate antigen 19-9; CEA, carcinoembryonic antigen; TBS, Tumor Burden Score; OS, Overall survival.

Bold values indicate p < 0.05.

**Table 4:** Univariable and multivariable analyses for RFS in the training cohort.

Variables	HR comparison	UV HR (95% CI)	UV *p* value	MV HR (95% CI)	MV *p* value
Age, year	≥60 *vs.* < 60	1.12 (0.91−1.37)	0.286		
Sex	Male *vs.* Female	0.84 (0.69−1.04)	0.107		
HBsAg	Positive *vs.* Negative	0.76 (0.63−0.93)	0.007	0.99(0.78−1.25)	0.932
Cirrhosis	Yes *vs.* No	0.79 (0.63−0.98)	0.030	0.85(0.67−1.08)	0.191
Child-Pugh grade	B *vs.* A	1.17 (0.72−1.90)	0.528		
NLR	>2.15 *vs.* ≤2.15	1.38 (1.12−1.70)	0.002	1.04(0.82−1.32)	0.719
PLR	>115.1 *vs.* ≤115.1	1.34 (1.10−1.63)	0.004	1.06(0.84−1.34)	0.638
PNI	>53.15 *vs.* ≤53.15	0.84 (0.68−1.04)	0.107		
GAR	>6.46 *vs.* ≤6.46	1.82 (1.44−2.29)	<0.001	1.33(1.03−1.70)	**0.028**
CEA, ng/mL	>5.0 *vs.* ≤5.0	1.57 (1.28−1.93)	<0.001	1.25(1.01−1.56)	**0.042**
CA19-9, U/mL	>37.0 *vs.* ≤37.0	1.82 (1.50−2.22)	<0.001	1.45(1.17−1.79)	**<0.001**
TBS, units	>6.0 *vs.* ≤6.0	1.79 (1.47−2.18)	<0.001	1.52(1.23−1.88)	**<0.001**
Anatomic resection	Yes *vs.* No	1.11 (0.89−1.40)	0.353		
Margin width, cm	≥1.0 *vs.* ≤1.0	0.80 (0.66−0.98)	0.032	0.96(0.78−1.18)	0.700
Macrovascular invasion	Yes *vs.* No	1.45 (1.11−1.91)	<0.001	1.26(0.95−1.66)	0.110
Microvascular invasion	Yes *vs.* No	1.49 (1.15−1.93)	0.003	1.20(0.91−1.57)	0.194
Satellite nodules	Yes *vs.* No	1.52 (1.22−1.89)	<0.001	1.22(0.97−1.53)	0.085
Tumor differentiation	Poor to moderate *vs.* Well	1.18 (0.77−1.81)	0.450		
Lymph node status	N1 *vs.* N0 + Nx	2.03 (1.63−2.53)	<0.001	1.63(1.29−2.06)	**<0.001**
Adjuvant chemotherapy	Yes *vs.* No	0.74 (0.55−1.01)	0.055		

HBsAg, Hepatitis B surface antigen; NLR, neutrophil-to-lymphocyte ratio; PLR, platelet-to-lymphocyte ratio; PNI, prognostic nutritional index; GAR, γ−glutamyl transferase to alanine aminotransferase ratio; CA19-9, carbohydrate antigen 19-9; CEA, carcinoembryonic antigen; TBS, Tumor Burden Score; RFS, Recurrence-free survival.

Bold values indicate p < 0.05.

### Effects of TCCA score on OS and RFS

**Figure 1:**
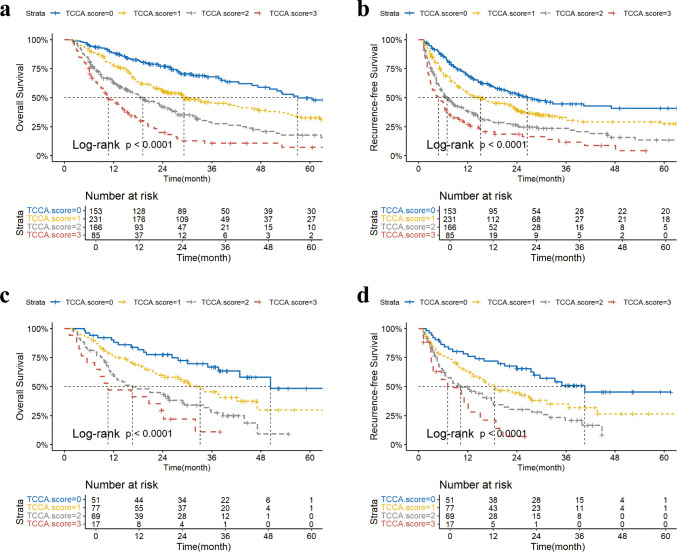
KM curves of overall survival (OS) and recurrence-free survival (RFS) of ICC patients stratified by TCCA model in the training cohort **(a, b)** and in the validation cohort **(c, d)**. TCCA score, Tumor Burden Score, carcinoembryonic antigen, and carbohydrate antigen 19–9 combined score.

At the end of the last follow-up, a total of 350 (55.1%) patients died and 400 (63.0%) patients recurred in the training cohort. Patients with higher TCCA score had an incremental worse OS (1−, 3−, and 5−year OS: score 0, 89%, 68%, 50%; *vs.* score 1, 79%, 46%, 33%; *vs.* score 2, 63%, 28%, 18%; *vs.* score 3, 48%, 12%, 8%; respectively, *p* < 0.001) and RFS (1−, 3−, and 5−year OS: score 0, 70%, 45%, 41%; *vs.* score 1, 54%, 33%, 28%; *vs.* score 2, 38%, 23%, 13%; *vs.* score 3, 26%, 12%, 3%; respectively, *p* < 0.001) (**Figures 1a, b**, **Supplementary Table S1**). In the validation cohort, Kaplan-Meier survival curves demonstrated significant differentiation in each group, with all *p* < 0.001 (**Figures 1c, d**, **Supplementary Table S1**).

### Comparison and validation of the TCCA model

**Figure 2:**
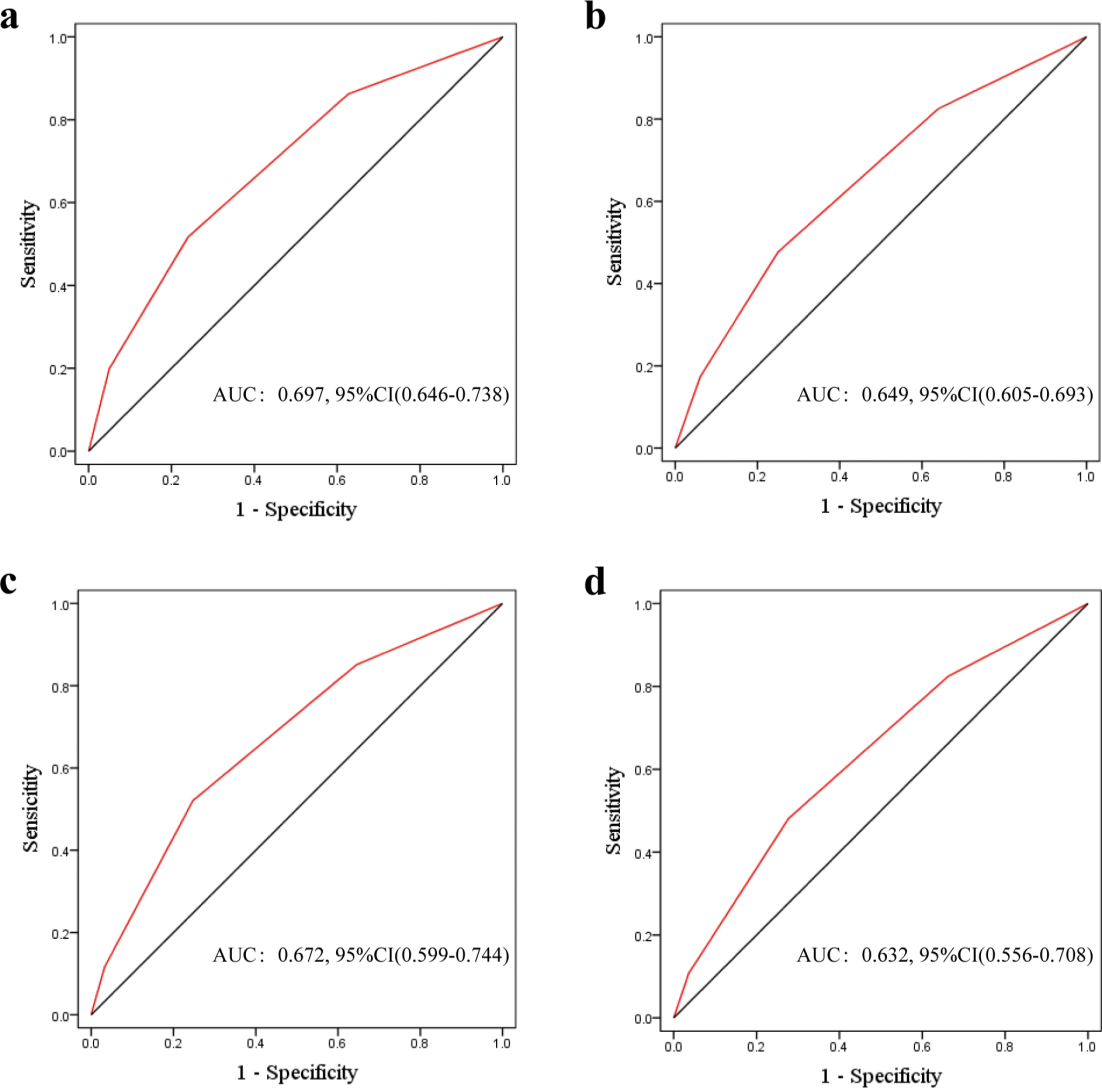
ROC curves of the TCCA model for overall survival (OS) and recurrence-free survival (RFS) in ICC Patients: Training **(a, b)** and Validation Cohorts **(c, d)**. TCCA, combination of Tumor Burden Score, carcinoembryonic antigen, and carbohydrate antigen 19-9.

The ROC curve of TCCA model showed an AUC value of 0.70 (95% CI = 0.67−0.73) for OS and 0.65 (95% CI = 0.61−0.69) for RFS in the training cohort (**Figures 2a, b**). In the validation cohort, TCCA model demonstrated moderate prognostic prediction capabilities (OS: AUC 0.67, 95% CI = 0.63−0.71; RFS: 0.63, 95% CI = 0.59−0.68) (**Figures 2c, d**). For calibration of the TCCA model, calibration plots depicted a good consistency between the predicted outcome and the observed outcome of the model in terms of 5−year OS and RFS in the training and validation cohorts (**Supplementary Figure S1**).

The TCCA model demonstrated superior discriminative ability for predicting outcomes, with thehighest C-index of 0.734 (95% CI: 0.711−0.757), compared to the AJCC 8^th^ TNMstaging system (C-index: 0.599), TBS-CEA score (C-index: 0.648), and TBS-CA19–9 score (C-index: 0.660). Additionally, the TCCA model yielded the lowest AIC value (3840.8), indicating a better model fit than the other systems. All comparisons were statistically significant (*p* < 0.001) (**Supplementary Table S2**).

### TCCA model performance in CEA- and CA19-9-negative subgroups

**Figure 3:**
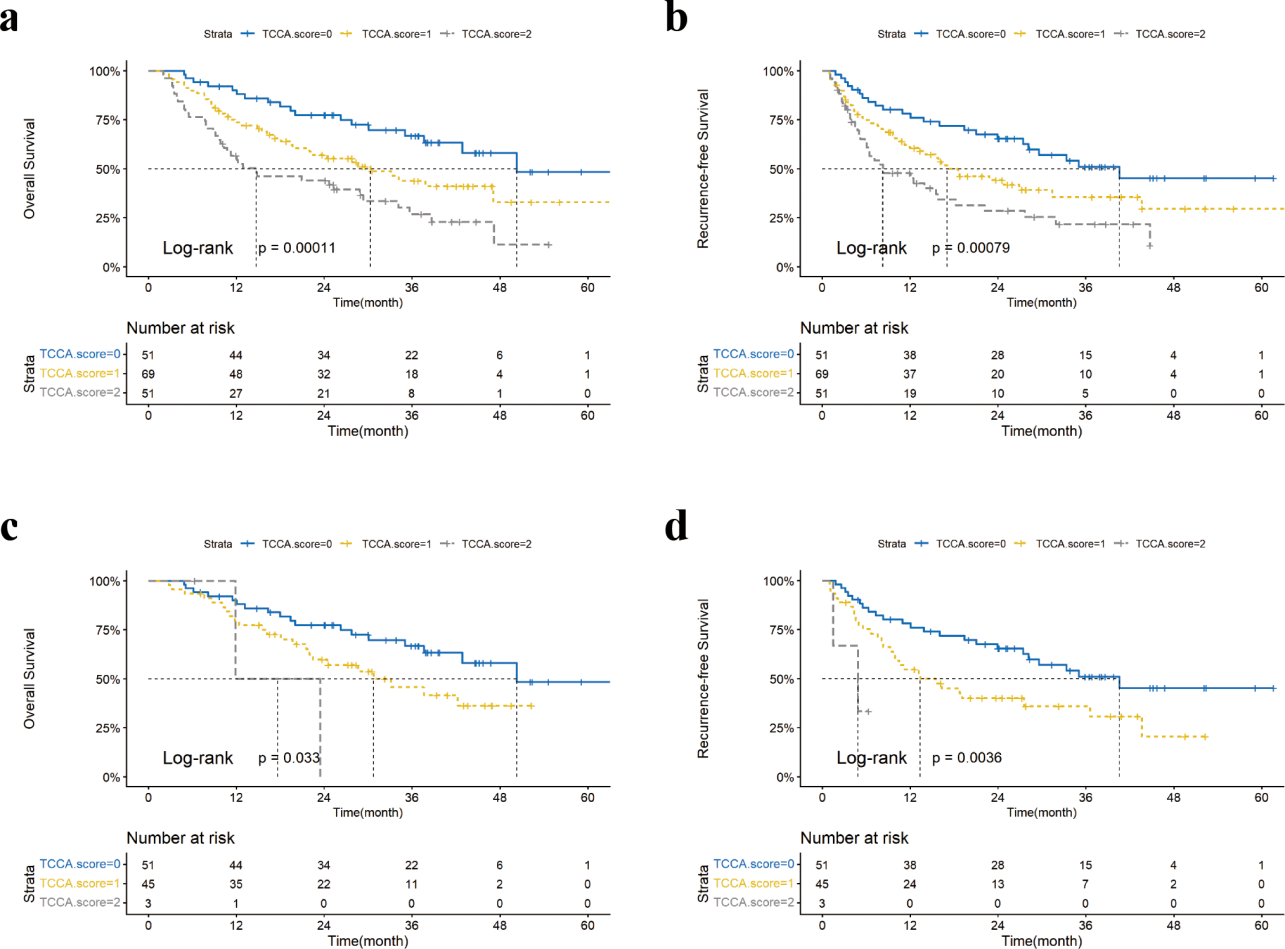
KM curves of overall survival (OS) and recurrence-free survival (RFS) of ICC patients stratified by the TCCA model in biomarker-negative subgroups of the validation cohort: Carcinoembryonic antigen (CEA)-negative subgroup **(a, b)** and Carbohydrate antigen 19-9 (CA19-9)-negative subgroup **(c, d)**. TCCA score, Tumor Burden Score, CEA, and CA19–9 combined score.

In the validation cohort, 80.0% and 46.3% of patients were negative for CEA and CA19-9, respectively. In the CEA-negative subgroup, the median OS for patients with TCCA scores of 0, 1, and 2 was 50.3, 30.3, and 14.8 months, respectively, while the median RFS were 40.6, 17.1, and 8.3 months (all *p* < 0.001) (**Figures 3a, b**). In the CA19-9-negative subgroup, the median OS for TCCA scores of 0, 1, and 2 was 50.3, 30.7, and 17.7 months (*p* = 0.033), and the median RFS were 40.6, 13.4, and 4.9 months (*p* = 0.004) (**Figures 3c, d**).

## Discussion

The proposed TCCA model represents a novel and comprehensive integration of tumor burden and tumor biology. While previous studies have linked TBS with either CA19–9 or CEA, the TCCA model is the first to combine all three preoperative indicators (TBS, CEA and CA19-9), reflecting both tumor morphology and biomarker-derived aggressiveness. This integration enhances prognostic accuracy, particularly among patients with negative CA19–9 or CEA levels, and reduces the risk of underestimating tumor aggressiveness when relying on a single biomarker. The TCCA model demonstrated superior discrimination and calibration performance (C-index 0.734) compared with previously established scores (TBS−CA19-9: 0.660; TBS−CEA: 0.648) and the AJCC−TNM staging system (0.599). In addition to its good prognostic performance, the TCCA model is simple and clinically practical, as it can be calculated preoperatively using routine imaging and serum data, enabling surgeons to provide individualized prognostic counseling during preoperative discussions.

The TCCA model incorporates three preoperative factors: TBS, CEA, and CA19-9, all of which were identified as independent risk factors for OS and RFS in the training cohort. Many studies have confirmed that both tumor size and tumor number are important prognostic factors, as reflected in the 8^th^ edition of AJCC T-staging system (20). However, these two parameters are categorized dichotomously with arbitrary cutoffs, which may limit their ability to assess prognosis accurately and provide personalized treatment recommendations for resectable ICC patients (21). TBS, a composite metric of tumor morphology and a continuous variable, may better reflect total tumor burden in relation to survival outcomes. The concept of TBS originates from the ‘Metro-ticket’ system, initially applied to patients with colorectal liver metastases, and was later shown to have a significant inverse relationship with OS in HCC patients undergoing liver transplantation (19, 22). Multivariate analysis in this study showed that high TBS was associated with a 1.68-fold increased risk of death (HR 1.68, 95% CI 1.35−2.11, *p* < 0.001) and a 1.52-fold increased risk of recurrence (HR 1.52, 95% CI 1.23−1.88, *p* < 0.001). In addition to tumor morphology, serum CEA and CA19–9 are recognized as surrogate markers of tumor biology and robust predictors of long-term outcomes in ICC patients (23). These well-established ICC biomarkers are widely used in clinical practice and can be easily assessed preoperatively. In our multivariate analysis, elevated levels of both CEA and CA19–9 were independently associated with worse OS and RFS, with elevated CEA levels linked to a 1.42-fold increased risk of death (HR 1.42, 95% CI 1.13−1.78, *p* = 0.003) and a 1.25-fold increased risk of recurrence (HR 1.25, 95% CI 1.01−1.56, *p* = 0.042), while elevated CA19–9 levels were associated with a 1.46-fold increased risk of death (HR 1.46, 95% CI 1.16−1.83, *p* = 0.001) and a 1.45-fold increased risk of recurrence (HR 1.45, 95% CI 1.17−1.79, *p* < 0.001). Our results demonstrate that all three variables—TBS, CEA, and CA19-9—serve as important prognostic indicators, providing valuable insights for predicting outcomes in ICC patients.

Currently, various preoperative models based on TBS have been developed, and the combination of TBS with other clinical variables exhibited enhanced predictive efficacy for patients following ICC resection. Munir et al. demonstrated an interplay between TBS and ALBI grade, revealing that patients with both high TBS and ALBI grade experienced significantly higher 2-year recurrence (84.6%) and 5-year mortality (94.6%) compared to those with both low TBS and ALBI grade (24). Similarly, Wang et al. constructed a novel index combining TBS with the albumin-to-alkaline phosphatase ratio (AAPR), which effectively stratified postoperative survival outcomes in ICC patients undergoing curative resection, particularly in predicting postoperative recurrence, with AUC values of 0.653 for OS and 0.658 for RFS (25). Additionally, Zhang et al. integrating TBS with more inflammatory and nutritional markers, such as AAPR, albumin–globulin ratio (AGR), and monocyte-to-lymphocyte ratio (MLR), to construct the TIIN score can improve predictive accuracy—with a 3-year OS AUC of 0.728 in the training cohort and 0.695 in the validation cohort—this approach also introduces greater complexity (26). Our multivariate analysis also identified GAR as an independent prognostic factor for OS and RFS. However, given that inflammatory and nutritional markers are highly susceptible to variations in infection status and nutritional status, limiting their capacity to accurately reflect tumor biology (27).

Incorporating tumor-specific biomarkers into prognostic models may be a better choice than relying on inflammatory and nutritional markers. CA19–9 and CEA are recommended as tumor markers for the early detection and diagnosis of ICC by the Chinese expert consensus on management of intrahepatic cholangiocarcinoma (2022 edition) and the Liver Cancer Study Group of Japan Clinical Practice Guidelines (28). Previous studies have developed prognostic models by combining TBS with CA19-9, consistently demonstrating that Elevated TBS and CA19–9 levels was associated with poor prognosis (18, 29). However, approximately 10% of the population are genotypically negative for Lewis blood group antigen and therefore unable to synthesize CA19-9, limiting its utility as a tumor marker in all patients (30). Integrating preoperative CEA into the prognostic models may enhance the stratification of postoperative prognosis in ICC patients.

The TCCA model, which integrates three preoperative variables—TBS, CEA, and CA19-9—was developed without the inclusion of commonly used postoperative pathological variables. Although our study confirmed that variables such as macrovascular invasion, satellite nodules, and lymph node metastasis are closely related to OS in multivariate analysis. Lymph node metastasis, in particular, is widely regarded as the strongest independent prognostic factor for long-term outcomes following ICC resection (31). Nevertheless, we believe that the TCCA model, by combining preoperative tumor morphology and biological markers, offers a sufficiently comprehensive reflection of the biological behavior of ICC. As shown in **Table 2**, higher TCCA scores were associated with a lower prevalence of HBsAg positivity, a known protective factor for ICC prognosis (32). Additionally, as TCCA scores increased, inflammatory markers such as NLR and GAR also increased incrementally, while the nutritional markers PNI decreased—trends that were all associated with poorer prognosis. Furthermore, higher TCCA scores were correlated with increased rates of macrovascular invasion, microvascular invasion, satellite nodules, and lymph node metastasis, suggesting more advanced tumor biology and worse outcomes. Thus, the TCCA model demonstrates correlations not only with inflammatory and nutritional markers but also with common postoperative pathological variables, underscoring its potential as a valuable tool for prognostic assessment.

The TCCA model offers several key advantages. First, it allows for the straightforward preoperative assessment of each parameter, as imaging and tumor biomarker testing are standard components of preoperative evaluation, and these markers are also routinely monitored during postoperative follow-up in ICC patients in China. Second, the cutoff values for TBS, CEA, and CA19–9 are clinically relevant and practical. The TBS cutoff was set at 6 units, and based on the Pythagorean theorem, it can be quickly deduced that any solitary tumor with a diameter of 6 cm or more will exceed this threshold, classifying the patient into the high TBS group. The cutoff values for CEA and CA19–9 were aligned with the upper limits of normal laboratory reference ranges, minimizing variability and potential errors associated with differing cutoff values across studies. Third, the TCCA model effectively stratifies patients based on OS and RFS, offering clinicians valuable guidance for preoperative decision-making. For patients with lower TCCA scores, more aggressive resection may be recommended to improve long-term outcomes, while those with higher scores could benefit more from postoperative adjuvant chemotherapy. This underscores the importance of preoperatively identifying high-risk patients to initiate timely adjuvant chemotherapy.

Several limitations must be considered when interpreting the data from this study. First, as with all retrospective studies, there is potential for selection bias in selecting patients for surgical resection. Second, different etiologies of ICC may lead to variations in CEA and CA19–9 levels. Given the study population was limited to China, where HBV infection rates are higher, further research is necessary to assess the applicability of this model to Western populations. Additional, CA19–9 is particularly influenced by biliary obstruction, and monitoring dynamic changes in these biomarkers may provide more accurate and reliable information (33). Finally, the AUC of the TCCA model ranged from 0.63 to 0.70, indicating that there remains room for improvement. Future studies may further enhance the model by integrating additional preoperative data, such as selected radiomic features or circulating tumor DNA, to better capture tumor heterogeneity and biological behavior (34).

## Conclusion

The TCCA model, which integrates tumor morphology and biological markers (TBS, CEA, and CA19-9), provides a reliable and practical preoperative tool for predicting survival outcomes in patients with ICC. Its simplicity and accessibility make it suitable for routine clinical application, assisting clinicians in risk stratification and optimization of treatment strategies for ICC patients before hepatectomy.

## Data Availability

The datasets used and/or analyzed during the current study are available from the corresponding author on reasonable request. Requests to access these datasets should be directed to Yongyi Zeng, lamp197311@126.com.

## Supplementary material

Supplementary Figure 1 Calibration curves for TCCA model to predict overall survival (OS) and recurrence-free survival (RFS) in training cohorts (a, b). TCCA, combination of Tumor Burden Score, carcinoembryonic antigen, and carbohydrate antigen 19-9.

Supplementary Table 1 OS and RFS data stratified by TCCA score in the training and validation cohort. Abbreviation:TCCA score, Tumor Burden Score, carcinoembryonic antigen, and carbohydrate antigen 19–9 combined score. OS, Overall survival; RFS, Recurrence-free survival.

Supplementary Table 2 Comparison of the TCCA score with AJCC staging system and other scoring systems in the training cohort. Abbreviation: TCCA score, Tumor Burden Score (TBS), carcinoembryonic antigen (CEA), and carbohydrate antigen 19-9 (CA19-9) combined score; TBS−CEA Score, TBS and CEA combined score; TBS−CA19–9 Score, TBS and CA19–9 combined score; CEA−CA19–9 Score, CEA and CA19–9 combined score; AJCC, American Joint Committee on Cancer; AIC: Akaike information criterion.
