# Supplementary figures and images for: Preoperative prognostic model combining tumor burden score and tumor markers to predict long-term outcomes following hepatectomy for intrahepatic cholangiocarcinoma: a multi-institutional analysis
Source: Front Oncol. 2026 Feb 11;16:1720482. doi: 10.3389/fonc.2026.1720482 (PMC12932142; doi:10.3389/fonc.2026.1720482)

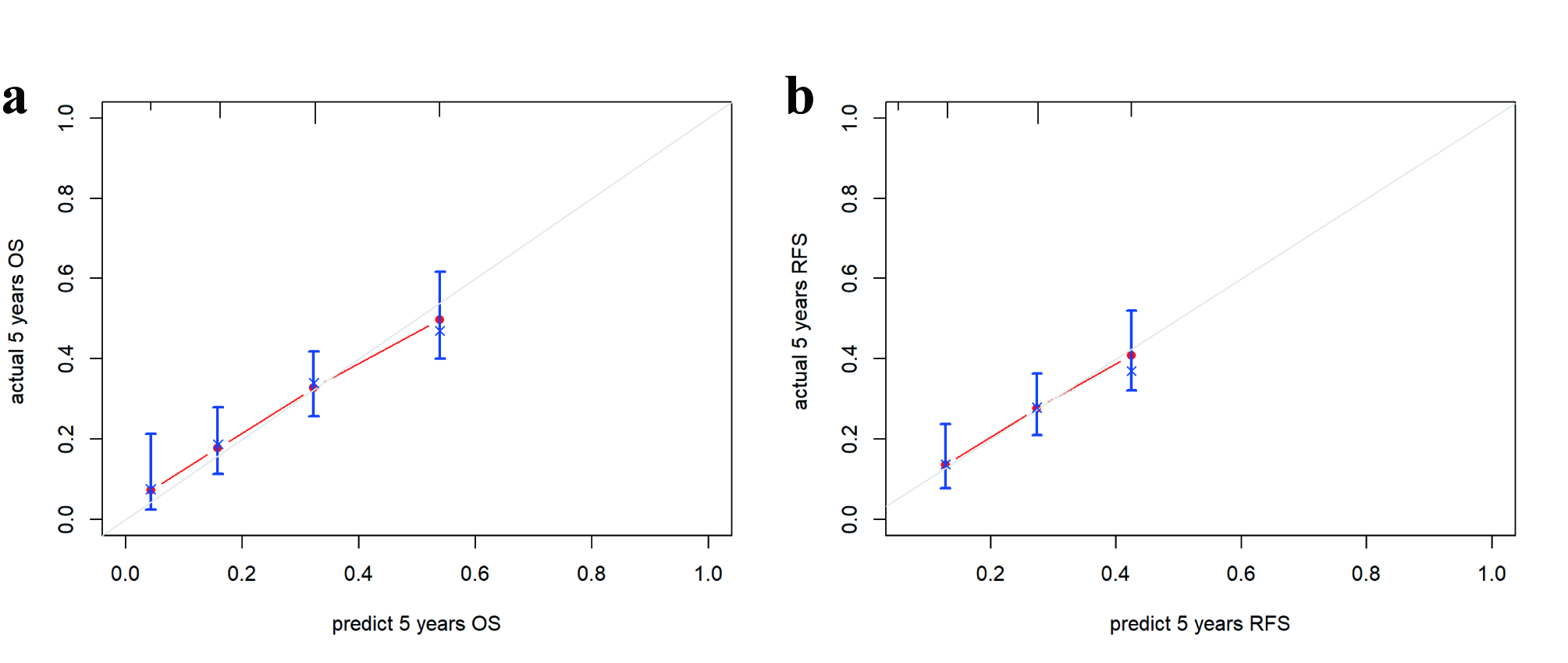

Supplement: Supplementary Figure 1 — Calibration curves for TCCA model to predict overall survival (OS) and recurrence-free survival (RFS) in training cohorts (a, b). TCCA, combination of Tumor Burden Score, carcinoembryonic antigen, and carbohydrate antigen 19-9. [file Image1.tif]
