# Supplementary material for: Preoperative prognostic model combining tumor burden score and tumor markers to predict long-term outcomes following hepatectomy for intrahepatic cholangiocarcinoma: a multi-institutional analysis
Source: Front Oncol. 2026 Feb 11;16:1720482. doi: 10.3389/fonc.2026.1720482 (PMC12932142; doi:10.3389/fonc.2026.1720482)
Supplement: Supplementary Table 1 — OS and RFS data stratified by TCCA score in the training and validation cohort. Abbreviation:TCCA score, Tumor Burden Score, carcinoembryonic antigen, and carbohydrate antigen 19–9 combined score. OS, Overall survival; RFS, Recurrence-free survival. [file DataSheet1.docx]

**Supplementary table 1. OS and RFS stratified by TCCA scores in the training and validation cohort**

| Cohort | TCCA score | OS | | | | RFS | | | |
| --- | --- | --- | --- | --- | --- | --- | --- | --- | --- |
|  |  | median, months | 1-year (%) | 3-year (%) | 5-year (%) | median, months | 1-year (%) | 3-year (%) | 5-year (%) |
| Training | 0 | 59.7 | 88.6 | 67.8 | 49.7 | 28.8 | 69.9 | 44.9 | 41.5 |
|  | 1 | 31.3 | 78.5 | 45.9 | 33.1 | 15.4 | 54.2 | 33.4 | 28.0 |
|  | 2 | 19.4 | 62.9 | 28.5 | 18.3 | 9.7 | 38.0 | 23.3 | 13.1 |
|  | 3 | 11.5 | 47.9 | 11.9 | 8.0 | 8.1 | 26.1 | 11.8 | 3.0 |
| Validation | 0 | 54.6 | 90.0 | 66.9 | 43.5 | 39.4 | 78.0 | 51.5 | 46.3 |
|  | 1 | 33.0 | 76.0 | 46.7 | 33.4 | 20.2 | 62.2 | 35.1 | 26.8 |
|  | 2 | 19.1 | 60.0 | 28.7 | 18.0 | 11.8 | 49.2 | 21.6 | 0 |
|  | 3 | 11.3 | 47.1 | 12.1 | 0 | 9.6 | 37.5 | 7.5 | 0 |

**Abbreviation**：TCCA, TBS, CEA, and CA19−9 combined score. OS, Overall survival; RFS, Recurrence-free survival.
