# Supplementary material for: Preoperative prognostic model combining tumor burden score and tumor markers to predict long-term outcomes following hepatectomy for intrahepatic cholangiocarcinoma: a multi-institutional analysis
Source: Front Oncol. 2026 Feb 11;16:1720482. doi: 10.3389/fonc.2026.1720482 (PMC12932142; doi:10.3389/fonc.2026.1720482)
Supplement: Supplementary Table 2 — Comparison of the TCCA score with AJCC staging system and other scoring systems in the training cohort. Abbreviation: TCCA score, Tumor Burden Score (TBS), carcinoembryonic antigen (CEA), and carbohydrate antigen 19-9 (CA19-9) combined score; TBS−CEA Score, TBS and CEA combined score; TBS−CA19–9 Score, TBS and CA19–9 combined score; CEA−CA19–9 Score, CEA and CA19–9 combined score; AJCC, American Joint Committee on Cancer; AIC: Akaike information criterion. [file DataSheet2.docx]

**Supplementary table 2. Comparison of the TCCA scoring system with AJCC staging system and other scoring systems in the training cohort**

| **Staging systems** | **C-index (95%CI)** | **Standard error** | ***p* value** | **AIC value** |
| --- | --- | --- | --- | --- |
| TCCA Score | 0.734 (0.711−0.757) | 0.012 | reference | 3840.8 |
| AJCC 8^th^ TNM Stage | 0.599 (0.568−0.630) | 0.016 | **<0.001** | 3980.1 |
| TBS - CEA Score | 0.648 (0.621−0.675) | 0.014 | **<0.001** | 3942.1 |
| TBS - CA19−9 Score | 0.660 (0.633−0.687) | 0.014 | **<0.001** | 3926.7 |
| CEA- CA19−9 Score | 0.623(0.594−0.652) | 0.015 | **<0.001** | 3959.9 |

**Abbreviation**: AJCC: American Joint Committee on Cancer; AIC: Akaike information criterion. Bole values indicate *p* ＜0.05.
